# Racial and Ethnic Differences in Falls Among Older Adults: a Systematic Review and Meta-analysis

**DOI:** 10.1007/s40615-021-01179-1

**Published:** 2021-11-16

**Authors:** Natasha Wehner-Hewson, Paul Watts, Richard Buscombe, Nicholas Bourne, David Hewson

**Affiliations:** 1 School of Health, Sport and Bioscience, University of East London, London, UK; 2 Institute for Health Research, University of Bedfordshire, University Square, Luton, LU1 3JU Bedfordshire UK

**Keywords:** Older adults, Ethnicity, Falls, Prevalence

## Abstract

The aim of this systematic review and meta-analysis was to determine whether differences in reported fall rates exist between different ethnic groups. Searches were carried out on four databases: Medline, Cumulative Index to Nursing and Allied Health Literature (CINAHL), Scopus, and Web of Science. Only English language studies with community-dwelling participants aged 60 + years were included. Studies also needed to compare fall prevalence for at least two or more ethnic groups. Two reviewers independently screened all articles and evaluated study quality. Twenty-three articles were included for systematic review, and meta-analyses were carried out on the 16 retrospective studies that reported falls in the previous 12 months. The Asian group demonstrated significantly lower fall prevalence than all other ethnic groups at 13.89% (10.87, 16.91). The Hispanic group had a fall prevalence of 18.54% (12.95, 24.13), closely followed by the Black group at 18.60% (13.27, 23.93). The White group had the highest prevalence at 23.77% (18.66, 28.88). Some studies provided adjusted estimates of effect statistics for the odds/risk of falls, which showed that differences still existed between some ethnic groups even after adjusting for other risk factors. Overall, differences in fall prevalence do appear to exist between different ethnic groups, although the reasons for these differences currently remain undetermined and require further investigation. These findings highlight the need to provide more ethnically tailored responses to public health challenges, which could potentially increase the adherence to prevention interventions, and allow for a more targeted use of resources.

## Introduction

Falls are one of the most common and most serious problems faced by older adults worldwide [1]. Falls can cause pain, injury and sometimes death, and can also have an impact on mental wellbeing for older adults, their family members and carers [2]. There are wide ranging and severe consequences of falls for both the individual that falls, and for health and care systems [3]. Injuries as the result of a fall range from abrasions and bruises, to hip fracture, with more serious injuries often resulting in institutionalisation [4], while 1 in 5 older adults die within 12 months of a hip fracture [5]. Evidence from systematic reviews of falls prevalence in community-based studies shows that the risk of falls is higher for women and with increasing age [6]. However, less is known about differences in the prevalence of falls between ethnic groups.

Substantial health inequalities exist between ethnic groups. In Europe for example, ethnic groups such as South Asians, Black Africans and Black Caribbeans experience higher rates of obesity, diabetes and cardiovascular disease, compared to White Europeans [7, 8]. These inequalities are due to underlying causal factors such as socio-economic factors, including lower levels of education, income, employment and even the built environment, although the contribution each factor plays, and exactly how they interact is difficult to determine. In addition, these inequalities often persist after controlling for socioeconomic disadvantage, suggesting that structural influences such as disparity and discrimination in access to health and social care [9], or cultural differences in behaviours or beliefs may be important factors [10].

Health inequalities occur across all age groups, but the greatest differences in health between ethnic groups are among older adults [11]. Health inequalities in older people are likely to increase due to population ageing in countries of all income groups [12]. People are now living for a considerable period in declining health, due to age associated health conditions such as frailty [13]. Falls in particular are likely to increase throughout ‘older age’ although it is not well understood how ethnic minorities are affected by life course health inequalities as they enter old age [14, 15].

The worldwide prevalence of falls is high, commonly reported as being a third for adults aged over sixty-five [16], increasing to 40% for those over eighty years of age [17]. However, the commonly reported fall prevalence of one-third is usually associated with studies carried out in Western countries, whereas other countries have reported differences in fall prevalence. For instance, China and Japan have noticeably lower reported fall rates than those seen in the West. A systematic review by Kwan et al. [18] reported a median fall prevalence of 18% in Chinese people from a sample of 21 studies. However, there have been very few studies looking at fall rates in pluricultural populations. Different ethnic groups within a country share common local cultural factors, while potentially differing in specific factors related to ethnicity. For example, within a community, obesity may be more prevalent in a particular ethnic group, even though all members of the community can be expected to be exposed to the same public health messaging about its risks via various media. This may be due to cultural attitudes to physical activity, food preferences, and body image [19].

This is particularly true for migrant groups [20]. In addition, studies that directly compare ethnic groups provide a homogenous methodology to each group, rather than different studies, using different methodologies looking at single ethnicities. The aim of this systematic review is therefore to determine whether differences in reported fall rates exist between different ethnic groups.

## Methods

### Search Strategy

The search was performed and reported following the Preferred Reporting Items for Systematic reviews and Meta-Analyses (PRISMA) [21]. Searches were carried out on the following databases: Medline, Cumulative Index to Nursing and Allied Health Literature (CINAHL), Scopus and Web of Science. Other relevant studies were also identified following individual searches of the reference lists in the articles selected. There was no limitation in publication date, and any articles that satisfied the search criteria were selected, up to the date of search, the end of December 2020. The Cochrane Population, Intervention, Comparison, Outcome (PICO) methodology was used to determine the keywords to be used in the search [22]. A summary of the PICO search strategy is shown in Table 1.

**Table 1:** PICO Search keywords and MeSH terms

PICO Term	Description	Keywords/MeSH	Search location
P–Population	Participants aged 60 +	Elder* OR older	Title/Abstract
		Aged	MeSH heading
	Community-dwelling	–	
	Ethnically or culturally homogenous population	Ethni* OR culture* OR rac*	Title/Abstract
I–Intervention	None	–	N/A
C–Comparison	Studies must include a comparison between two or more ethnic/cultural/racial groups	–	N/A
O–Outcome(s)	Primary: fall prevalence	Fall*	Title/Abstract
		Fall	MeSH heading
	Secondary: Fall with injury prevalence	–	N/A
T–Time	Unlimited	–	N/A
S–Study design	Any quantitative study	–	N/A

### Selection Criteria

This review included studies of community-dwelling participants, while studies including institutionalised people (hospitals, care homes…) were excluded. All participants were aged 60 + years, and any studies including younger participants were excluded. To be included, studies needed to provide results separately either for all ethnic groups in the same country, or the same ethnic group in multiple countries. Studies where ethnic identity was not specified, contained mixed ethnic groups, groups titled ‘other’, or had only single ethnic groups with no comparison to others, were excluded. Studies needed to report fall prevalence, either as number of falls, rate of falls or number of participants who experienced at least one fall, to be included. Only studies written in English were included.

### Data Extraction

Keyword searches were carried out on all four databases. The results were imported into EndNote X9 (Clarivate Analytics, Philadelphia, PA, USA), and all duplicates were removed. Titles and abstracts were reviewed by two researchers to determine relevant studies. Full text versions of each paper were obtained for detailed review and extraction of data. Selected data from each study were entered on an Excel template, with extracted data including participant demographics such as age, ethnicity, country of study, living situation, whether the group was ethnically homogeneous, comparison of two or more ethnic groups, fall prevalence and study design. Selected studies were critically assessed using the ‘Quality Assessment Tool for Observational Cohort and Cross-Sectional Studies’ [23]. Fourteen questions were answered as ‘yes’, ‘no’ or ‘Other (cannot be determined, not applicable, not reported)’. Two reviewers assessed all articles independently, and any disagreements were resolved following discussion with a third party. A score was generated as a percentage, without considering any ‘not applicable’ responses. Scores rated < 50% were considered to be ‘poor’, with 50–74% considered to be ‘fair’, while those rated ≥ 75% considered to be of ‘good’ quality.

### Meta-Analysis

Following the systematic review, quantitative meta-analysis was carried out in order to provide an overall fall prevalence for the largest groups present in the literature. The different ethnic groups were combined, where possible, under four general headings: Asian (including Asian, Chinese, Filipino and Japanese), Black (including African-American, Afro-Caribbean, Black, and Black-African), Hispanic (including Latino and Hispanic) and White (including Australian-born Australian, Caucasian, European-American, Italian-born Australian and Non-Hispanic White). These groups were chosen based on the NIH definitions for racial and ethnic categories [24].

The heterogeneity of the selected studies was evaluated using the *I*^2^ statistic, with boundaries of 25%, 50% and 75% taken to represent low, moderate and high heterogeneity, respectively [25]. Due to the high heterogeneity found across the studies with a fixed model, a random effects model was used for all meta-analyses. The meta-analysis was performed using a Microsoft Excel spreadsheet adapted from Neyeloff et al. [26]. Fall prevalence rates were weighted across ethnic groups using the inverse variance for each study. Data were reported as mean prevalence rates and 95% confidence intervals, with statistical significance taken to be *p* < 0.05. Forest plots were used to visualize the distribution of the fall prevalence data from the different studies included.

## Results

### Article Selection

**Fig. 1:**
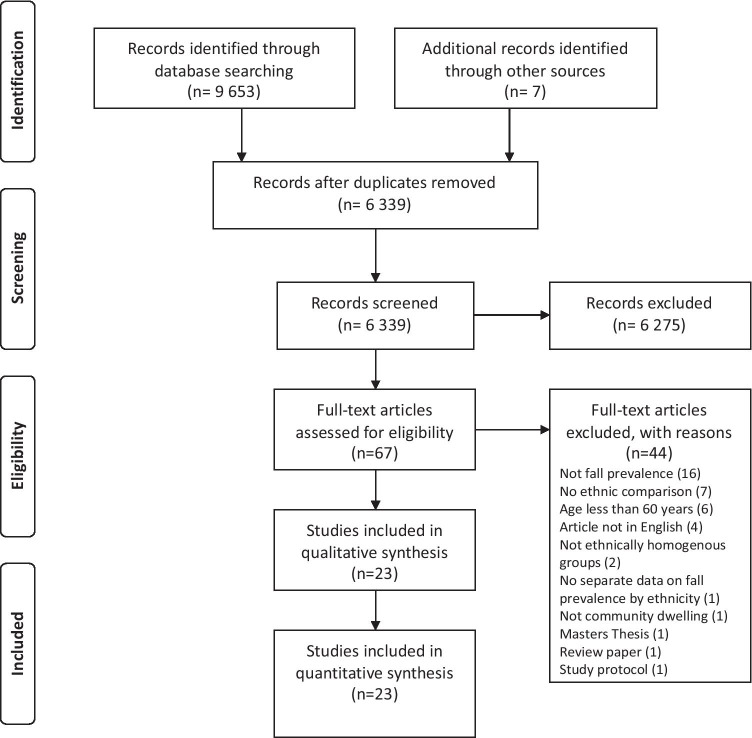
PRISMA flowchart of the article selection process [21]

The article selection PRISMA flowchart for this systematic review is included in Fig. 1. A total of 9 653 articles was identified during the database searches, which decreased to 6339 following removal of duplicates. After title and abstract screening, 6272 articles were removed leaving 67 articles for full-text appraisal. A further 44 articles were excluded due to reasons including lack of ethnic comparison, the inclusion of participants under the age of 60, non-English language articles, ethnic groups that were not homogeneous or participants who were not community-dwelling. The final selection consisted of 23 articles, the characteristics of which are shown in Table 2, including quality appraisal scores.

**Table 2:** Characteristics of selected articles

Authors	Country	Ethnic group	Age	Fall reporting	Quality score (%)
Aoyagi et al. (1998) [27]	Japan	Japanese	≥ 6 years	Retrospective 12 months	60.0
	USA	Japanese			
Chan et al. (1997) [28]	Singapore	Chinese, Indian, Malay	≥ 60 years	Retrospective 12 months	60.0
Chen et al. (2018) [29]	Singapore	Chinese, Indian, Malay	≥ 65 years	Retrospective 12 months	87.5
Davis et al. (1999) [30]	USA mainland	White	≥ 65 years	Prospective 24 months	90.0
	Hawaii	Japanese	≥ 65 years		
de Rekeneire et al. (2003) [31]	USA	Black, White	70–79 years	Retrospective 12 months	87.5
El Fakiri et al. (2018) [32]	The Netherlands	White, Moroccan, Surinamese, Turkish	≥ 65 years	Retrospective 12 months	70.0
Faulkner et al. (2005) [33]	USA	Black, White	≥ 65 years	Prospective every 4 months for up to 5.7 years	88.9
Geng et al. (2017) [34]	USA	Asian, Black, Hispanic, White	65–90 years	Retrospective 12 months	80.0
Hanlon et al. (2002) [35]	USA	Black, White	≥ 65 years	Retrospective 12 months	90.0
Kalula et al. (2015) [36]	South Africa	Black African, White	≥ 65 years	Retrospective 12 months	72.7
Karter et al. (2015) [37]	USA	Asian, Black, Filipino, Hispanic, White	≥ 60 years	EMR data only	100.0
Kwan et al. (2013) [38]	Hong Kong	Chinese	≥ 65 years	Prospective 12 months	100.0
	Taiwan	Chinese		Prospective 24 months	
	Australia	Chinese		Prospective 12 months	
	Australia	White		Prospective 12 months	
Kwon et al. (2018)	USA	Asian, Black, Hispanic, White	≥ 65 years	Retrospective 12 months	77.8
Leong Joyce et al. (2020) [39]	Malaysia	Chinese, Indian, Malay	≥ 60 years	Retrospective 12 months	80.0
Means et al. (2000) [40]	USA	Black, White	≥ 65 years	Retrospective 12 months	66.7
Nicklett and Taylor (2014) [41]	USA	Black, Hispanic, White	≥ 65 years	Retrospective 24 months	90.0
Qin and Baccaglini (2016) [42]	USA	Asian, Black, Hispanic, White	≥ 65 years	Retrospective 12 months	87.5
Sampaio et al. (2013) [43]	Brazil	Brazilian	≥ 65 years	Retrospective 12 months	70.0
	Japan	Japanese			
Stanaway et al. (2011) [44]	Australia	Australian-born Australian, Italian-born Australian	≥ 70 years	Retrospective 12 months, followed by prospective every 4 months for 4–40 months	90.9
Stevens et al. (2008) [45]	USA	American Indian/Alaskan Native, Black, Hispanic, White	≥ 65 years	Estimated data	75.0
Sun et al. (2016) [46]	USA	Black, White	≥ 65 years	Retrospective 12 months	90.9
Vieira et al. (2015) [47]	USA	African-American, Afro-Caribbean, European-American, Hispanic	≥ 60 years	Retrospective 24 months	77.8
Yeong et al. (2016) [48]	Malaysia	Chinese, Indian, Malay, Indigenous	≥ 60 years	Retrospective 12 months	77.8

### Article Description

The selected articles included 5,727,024 participants overall, with study sample sizes ranging from 114 [43] to 5,519,341 [45]. Studies were conducted with many different ethnic groups in several countries. There were 13 studies in the USA; two studies in Australia, Japan, Malaysia and Singapore; and 1 study from Brazil, Hong Kong, The Netherlands, South Africa and Taiwan. The 23 articles included nineteen retrospective studies, three prospective studies and one Electronic Medical Record study. Of the retrospective studies, 16 reported falls in the previous 12 months, two reported falls in the previous 24 months, while one study looked at falls in the previous 3 months.

### Quality Assessment

The quality appraisal scores ranged from 60 to 100% of the maximum score for each article. Of the 23 studies included, 6 were rated as fair, with the remaining 17 articles rated as good.

### Fall Prevalence

Fall prevalence was reported for 22 of the 23 studies and is shown in Table 3. Prevalence varied widely across the studies, from 2.9% (95% CI: 0.1, 5.6) for Chinese people in Malaysia [48], to 44.5% (95% CI: 37.8, 51.2) for Malays in Malaysia [39].

**Table 3:** Prevalence of falls

Authors	Country	Ethnic group	Sample size	Type of fall	Fall prevalence (%)	95% Confidence Interval
Aoyagi et al. (1998) [27]	Japan	Japanese men	624	Single fall	9.5%	(7.2, 11.8)
	Japan	Japanese-American men	436	Single fall	11.5%	(8.4, 14.5)
	USA	Japanese-American women	618	Single fall	16.8%	(13.9, 19.8)
	USA	Japanese women	910	Single fall	19.1%	(16.6, 21.7)
Chan et al. (1997) [28]	Singapore	Indian	24	Single fall	4.2%	(0.0, 12.2)
	Singapore	Chinese	333	Single fall	17.1%	(13.1, 21.2)
	Singapore	Malay	31	Single fall	35.5%	(18.6, 52.3)
Chen et al. (2018) [29]	Singapore	Malay	327	Injurious	4.6%	(2.3, 6.9)
	Singapore	Chinese	1446	Injurious	4.8%	93.7, 5.9)
	Singapore	Indian	202	Injurious	6.4%	(3.1, 9.8)
	Singapore	Chinese	1446	Single fall	11.7%	(10.0, 13.3)
	Singapore	Malay	327	Single fall	17.4%	(13.3, 21.5)
	Singapore	Indian	202	Single fall	20.8%	(15.2, 26.4)
de Rekeneire et al. (2003) [31]	USA	Black	1270	Single fall	18.8%	(16.7, 21.0)
	USA	White	1780	Single fall	23.2%	(21.2, 25.2)
El Fakiri et al. (2018) [32]	The Netherlands	White	7952	Recurrent falls	13.1%	(12.4, 13.9)
	The Netherlands	Moroccan	165	Recurrent falls	17.0%	(11.2, 22.7)
	The Netherlands	Surinamese	587	Recurrent falls	21.0%	(17.7, 24.2)
	The Netherlands	Moroccan	165	Single fall	30.3%	(23.3, 37.3)
	The Netherlands	Turkish	188	Recurrent falls	20.7%	(14.9, 26.5)
	The Netherlands	White	7952	Single fall	32.5%	(31.5, 33.5)
	The Netherlands	Surinamese	587	Single fall	37.1%	(33.2, 41.0)
	The Netherlands	Turkish	188	Single fall	32.4%	(25.8, 39.1)
Faulkner et al. (2005) [33]	USA	Caucasian	1665	Single fall	24.7%	(22.6, 26.8)
	USA	Black	156	Single fall	27.6%	(20.6, 34.6)
Geng et al. (2017) [34]	USA	Asian	684	Single fall	20.0%	(17.0, 23.0)
	USA	Black	463	Single fall	23.3%	(19.5, 27.2)
	USA	Hispanic	425	Single fall	27.8%	(23.5, 32.0)
	USA	White	4705	Single fall	28.5%	(27.2, 29.8)
Hanlon et al. (2002) [35]	USA	Black	1049	Single fall	20.2%	(17.8, 22.6)
	USA	White	1947	Single fall	23.2%	(21.3, 25.1)
Kalula et al. (2015) [36]	South Africa	Black African	283	Single fall	6.4%	(0.0, 14.6)
	South Africa	White	140	Single fall	42.9%	(40.0, 45.7)
Karter et al. (2015) [37]	USA	Filipino	8162	Single fall	3.7%	(3.3, 4.1)
	USA	Asian	11,275	Single fall	5.3%	(4.9, 5.7)
	USA	Black	11,417	Single fall	5.7%	(5.3, 6.2)
	USA	Latino	14,324	Single fall	6.8%	(6.4, 7.2)
	USA	Non-Hispanic White	63,509	Single fall	8.5%	(8.3, 8.7)
Kwan et al. (2013) [38]	Hong Kong	Chinese	201	Single fall	26.4%	(21.2, 31.5)
	Taiwan	Chinese	280	Single fall	28.9%	(22.8, 35.0)
	Australia	Chinese	211	Single fall	28.9%	(22.8, 35.0)
	Australia	White	764	Single fall	32.1%	(29.4, 34.7)
Kwon et al. (2018)	USA	Asian	1199	Recurrent falls	7.6%	(6.1, 9.1)
	USA	White	10,527	Recurrent falls	12.8%	(12.2, 13.4)
	USA	Hispanic	1423	Recurrent falls	14.8%	(13.0, 16.7)
	USA	Black	595	Recurrent falls	14.1%	(11.3, 16.9)
Leong Joyce et al. (2020) [39]	Malaysia	Malay	209	Single fall	44.5%	(37.8, 51.2)
	Malaysia	Chinese	49	Single fall	34.7%	(21.4, 48.0)
	Malaysia	Indian	50	Single fall	14.0%	(4.4, 23.6)
Means et al. (2000) [40]	USA	Black	118	Single fall	32.2%	(23.8, 40.6)
	USA	White	180	Single fall	32.8%	(25.9, 39.6)
Nicklett and Taylor (2014) [41]	USA	Black	1326	Single fall	26.8%	(24.4, 29.2)
	USA	White	8429	Single fall	29.2%	(28.2, 30.2)
	USA	Hispanic	729	Single fall	31.6%	(28.2, 34.9)
Qin and Baccaglini (2016) [42]	USA	Black	583	Recurrent falls	9.8%	(7.4, 12.2)
	USA	Asian	1193	Recurrent falls	10.1%	(8.4, 11.9)
	USA	White	10,359	Recurrent falls	13.0%	(12.3, 13.6)
	USA	Hispanic	1395	Recurrent falls	14.3%	(12.5, 16.2)
Sampaio et al. (2013) [43]	Brazil	Brazilian	74	Single fall	27.0%	(16.9, 37.1)
	Japan	Japanese	40	Single fall	32.5%	(18.0, 47.0)
Stanaway et al. (2011) [44]	Australia	Italian-born Australian	335	Recurrent falls	11.3%	(7.9, 14.7)
	Australia	Australian-born Australian	848	Recurrent falls	22.4%	(19.6, 25.2)
Stevens et al. (2008) [45]	USA	Black	346,155	Single fall	13.0%	(12.9, 13.1)
	USA	White	4,643,692	Single fall	15.8%	(15.8, 15.8)
	USA	Hispanic	457,096	Single fall	17.4%	(17.3, 17.5)
		American Indian/Alaskan				
	USA	Native	72,398	Single fall	27.8%	(27.5, 28.1)
Sun et al. (2016) [46]	USA	Black	1662	Single fall	27.1%	(24.9, 29.2)
	USA	White	5186	Single fall	33.8%	(32.5, 35.1)
Vieira et al. (2015) [47]	USA	Afro-Caribbean	109	Single fall	23.9%	(15.9, 31.9)
	USA	European-American	222	Single fall	38.7%	(32.3, 45.1)
	USA	Hispanic	113	Single fall	38.9%	(29.9, 47.9)
	USA	African-American	106	Single fall	39.6%	(30.3, 48.9)
Yeong et al. (2016) [48]	Malaysia	Chinese	140	Single fall	2.9%	(0.1, 5.6)
	Malaysia	Indian	28	Single fall	3.6%	(0.0, 10.4)
	Malaysia	Malay	631	Single fall	4.1%	(2.6, 5.7)

**Fig. 2:**
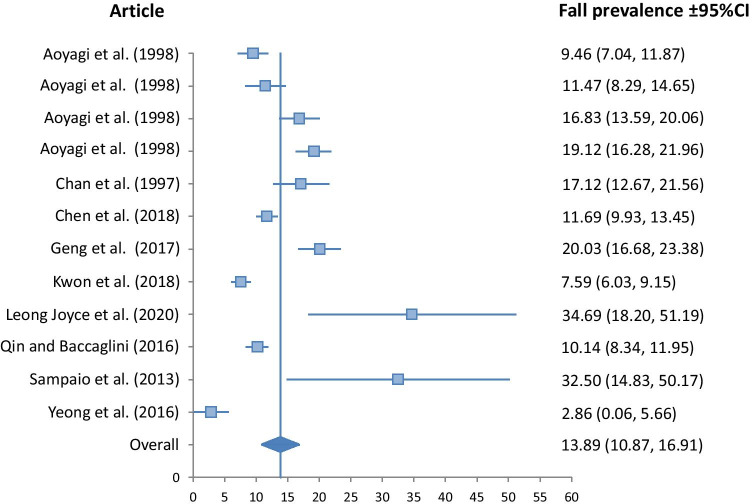
Fall prevalence for Asian ethnicity (*I*^2^*v* = 32.02, *p* < 0.001, *Qv* = 22.07)

**Fig. 3:**
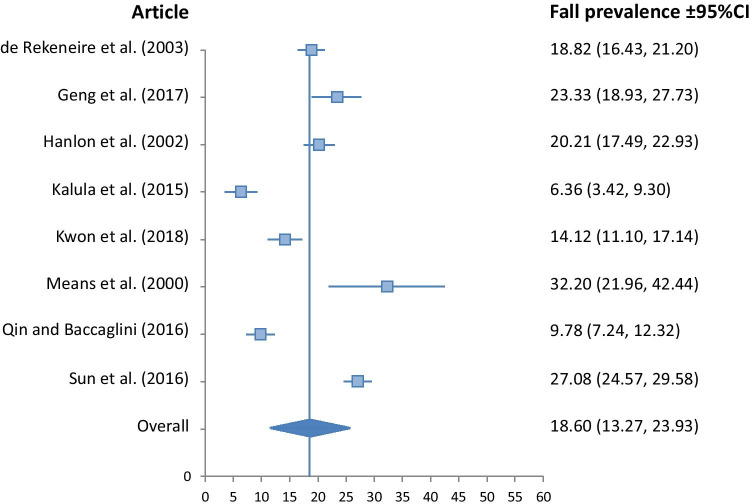
Fall prevalence for Black ethnicity (*I*^2^*v* = 17.57, *p* < 0.001, *Qv* = 8.49)

**Fig. 4:**
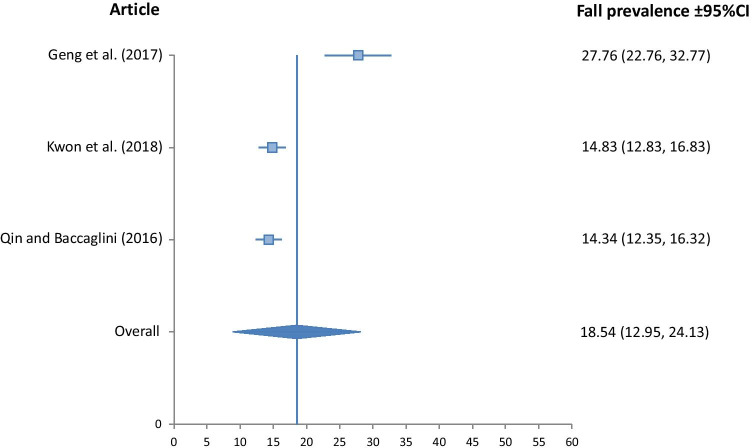
Fall prevalence for Hispanic ethnicity (*I*^2^*v* = 55.49, *p* < 0.001, *Qv* = 4.49)

**Fig. 5:**
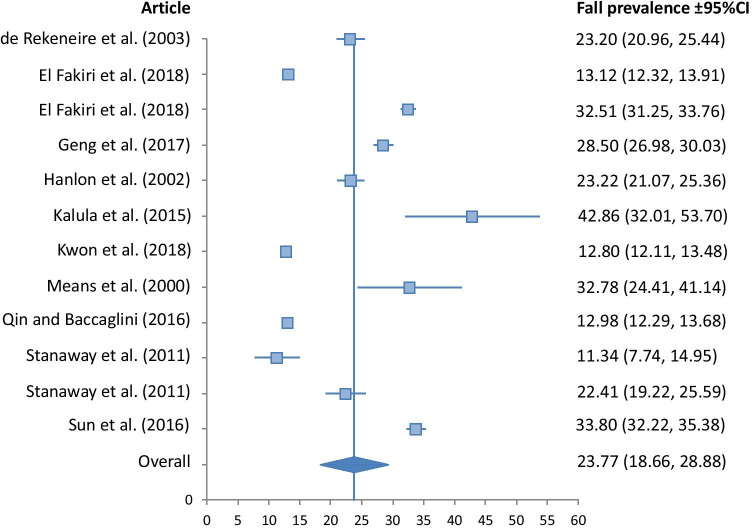
Fall prevalence for White ethnicity (*I*^2^*v* = 18.96, *p* < 0.001, *Qv* = 13.57)

A meta-analysis of fall prevalence was undertaken only for those 16 retrospective studies that reported falls in the previous 12 months, with Forest Plots shown in Figs. 2, 3, 4 and 5.

The Asian group demonstrated significantly lower fall prevalence than all other ethnic groups at 13.89% (10.87, 16.91). The Hispanic group had a fall prevalence of 18.54% (12.95, 24.13), closely followed by the Black group at 18.60% (13.27, 23.93). The White group had the highest prevalence at 23.77% (18.66, 28.88). Heterogeneity of studies included in the meta-analysis was low for the Black, and White groups, with *I*^2^_v_ measures of 17.57, and 18.96 respectively. It was moderate for the Asian group at 32.02, and high for the Hispanic group at 55.49.

### Fall Risk

#### Unadjusted Odds Ratios/Relative Risk

Most studies included comparisons with white participants (seven studies in the USA, one in Australia and one in South Africa), with only a few comparing fall prevalence with other ethnic groups. The unadjusted effect statistics of these comparisons for single falls are shown in Table 4. Overall results followed those of the fall prevalence meta-analysis, suggesting that White older adults tend to fall more than other ethnic groups (Black, Asian, Hispanic, Caribbean, Japanese, Filipino). There was some evidence of other differences in Asian countries, but the results were variable.

**Table 4:** Unadjusted odds ratios/relative risk

Authors	Ethnic group	Gender	Sample size	Effect size
Aoyagi et al. (1998) [27]	Japanese (Japan)	Male	624	-
	Japanese (Hawaii)	Male	436	1.1 (0.7, 1.6)
	Japanese (Japan)	Female	910	-
	Japanese (Hawaii)	Female	618	0.8 (0.6, 1.1)
Chan et al. (2017) [28]	Chinese (Singapore)	Male & Female	333	-
	Malay (Singapore)	Male & Female	31	2.66 (1.21, 5.86)*
	Indian (Singapore)	Male & Female	24	0.21 (0.03, 1.59)
Chen et al. (2018) [29]	Chinese (Singapore)	Male & Female	1446	-
	Malay (Singapore)	Male & Female	327	1.45 (1.05, 2.00)*
	Indian (Singapore)	Male & Female	202	2.01 (1.40, 2.88)*
Davis et al. (1999) [30]	Japanese (Hawaii)	Female	690	-
	White (USA)	Female	9689	1.8 (1.6, 2.0)*
Faulkner et al. (2005) [33]	White (USA)	Female	1665	-
	Black (USA)	Female	156	1.17 (0.78, 1.75) §
Geng et al. (2017) [34]	White (USA)	Female	4705	-
	Hispanic (USA)	Female	425	0.97 (0.74, 1.27)
	Black (USA)	Female	463	0.77 (0.59, 1.00)
	Asian (USA)	Female	684	0.63 (0.50, 0.80)*
Hanlon et al. (2002) [35]	White (USA)	Male & Female	1947	-
	Black (USA)	Male & Female	1049	0.77 (0.62, 0.94)*
Kalula et al. (2015) [36]	Black (South Africa)	Male & Female	283	-
	White (South Africa)	Male & Female	140	1.04 (1.01, 1.08)*
Karter et al. (2015) [37]	White (USA)	Male & Female	63,509	-
	Black (USA)	Male & Female	11,417	0.64 (0.59, 0.70) §*
	Asian (USA)	Male & Female	11,275	0.65 (0.59, 0.71) §*
	Filipino (USA)	Male & Female	8162	0.49 (0.44, 0.56) §*
	Hispanic (USA)	Male & Female	14,324	0.84 (0.78, 0.90) §*
Kwan et al. (2013) [38]	White (Austalia)	Male & Female	764	-
	Chinese (Taiwan)	Male & Female	280	0.39 (0.3, 0.49) §*
	Chinese (Hong Kong)	Male & Female	201	0.28 (0.19, 0.41) §*
	Chinese (Australia)	Male & Female	211	0.5 (0.37, 0.67) §*
Sun et al. (2016) [46]	White (USA)	Male & Female	5186	-
	Black (USA)	Male & Female	1662	0.7 (0.6, 0.8) §*
Vieira et al. (2015) [47]	Afro-Caribbean (USA)	Male & Female	222	-
	White (USA)	Male & Female	109	1.57 (1.08, 2.29) §*
	African-American (USA)	Male & Female	106	1.63 (1.07, 2.47) §*
	Hispanic (USA)	Male & Female	113	1.62 (1.07, 2.44) §*
Yeong et al. (2016) [48]	Malay (Malaysia)	Male & Female	631	-
	Chinese (Malaysia)	Male & Female	140	0.68 (0.24, 1.99)
	Indian (Malaysia)	Male & Female	28	0.86 (0.11, 6.59)
	Indigenous (Malaysia)	Male & Female	12	4.65 (0.97, 22.33)

Results are listed as Odds Ratio unless specified, § Relative Risk

* significantly different from reference group (*p* < 0.05)

#### Adjusted Odds Ratios/Relative Risk

Some studies provided adjusted estimates of effect statistics for the odds/risk of falls. These adjustments included a range of factors such as co-morbidities, depression, mobility limitations, functional tests and sociodemographic characteristics. These adjusted effect statistics are shown for single falls in Table 5, and recurrent falls in Table 6.

**Table 5:** Adjusted odds ratios/relative risk (single falls)

Authors	Ethnic group	Gender	Sample size	Effect size	Covariates
Chen et al. (2018) [29]	Chinese (Singapore)	Male & Female	1446	-	Age, sex, marrital status, cognitive function, self-reported pain, comorbidities, depression, BMI, difficulties with ADL, social network, mobility difficulties, grip strength
	Malay (Singapore)	Male & Female	327	4.76 (1.21, 18.68)*	
	Indian (Singapore)	Male & Female	202	4.50 (0.73, 27.64)	
Davis et al. (1999) [30]	Japanese (Hawaii)	Female	690	-	Age, height, weight, functional tests
	White (USA)	Female	9689	1.8 (1.5, 2.1)*	
de Rekeneire et al. (2003) [31]	Black (USA)	Male & Female	1270	-	Age, race, study site, BMI
	White (USA)	Male & Female	1780	1.4 (1.2, 1.6)*	
Faulkner et al. (2005) [33]	White (USA)	Female	1665	-	Grip strength, number of chronic conditions, and depression
	Black (USA)	Female	156	1.20 (0.80, 1.81) §	
Geng et al. (2017) [34]	White (USA)	Female	4705	-	Age, co-morbidities, poor health, and mobility limitations
	Hispanic (USA)	Female	425	0.94 (0.71, 1.24)	
	Black (USA)	Female	463	0.73 (0.55, 0.95)*	
	Asian (USA)	Female	684	0.64 (0.5, 0.81)*	
Kwan et al. (2013) [38]	White (Austalia)	Male & Female	764	-	Age, sex, incontinence, Parkinson's, education, FES-I
	Chinese (Taiwan)	Male & Female	280	0.98 (0.45, 2.11) §	
	Chinese (Hong Kong)	Male & Female	201	0.55 (0.17, 1.79) §	
	Chinese (Australia)	Male & Female	211	0.6 (0.23, 1.59) §	
Nicklett and Taylor (2014) [41]	White (USA)	Male & Female	8429	-	Adjusted for sociodemographic and health characteristics
	Black (USA)	Male & Female	1326	0.65 (0.53, 0.80)*	
	Hispanic (USA)	Male & Female	729	0.91 (0.69, 1.20)	
Yeong et al. (2016) [48]	Malay (Malaysia)	Male & Female	631	-	Age, sex, total income, physical activity level, living alone, number of co-morbidities, number of medications
	Chinese (Malaysia)	Male & Female	140	0.61 (0.2, 1.86)	
	Indian (Malaysia)	Male & Female	28	0.77 (0.1, 6.16)	
	Indigenous (Malaysia)	Male & Female	12	6.06 (1.10, 33.55)*	

Results are listed as Odds Ratio unless specified otherwise; § Relative Risk, * significantly different from reference group (*p* < 0.05)

Activities of daily living (ADL), Body mass index (BMI), Falls efficacy scale- International (FES-I)

**Table 6:** Adjusted odds ratios (recurrent falls)

Authors	Ethnic group	Gender	Sample size	Effect size	Covariates
El Fakiri et al. (2018) [32]	White (Netherlands)	Male & Female	7952	-	Age, sex, education, income, deprived neighbourhood, living alone, health (overweight, inactivity, alcohol, perecived health, hearing, sight, mobility limitations, multi-morbidity, loneliness, depression)
	Moroccan (Netherlands)	Male & Female	165	0.54 (0.27, 1.06)	
	Turkish (Netherlands)	Male & Female	188	0.84 (0.42, 1.64)	
	Surinamese (Netherlands)	Male & Female	587	1.05 (0.68, 1.64)	
Kwon et al. (2018)	White (USA)	Male & Female	10,527	-	Age, sex, marital status, poverty, BMI, chronic diseases, functional limitation
	Black (USA)	Male & Female	595	0.82 (0.51, 1.30)	
	Asian (USA)	Male & Female	1199	0.63 (0.43, 0.92)*	
	Hispanic (USA)	Male & Female	1423	0.98 (0.72, 1.34)	

Results are listed as Odds Ratio, * significantly different from reference group (*p* < 0.05)

Body mass index (BMI)

These data show differences in the odds/risk of falling still existed between some ethnic groups even after adjusting for other risk factors. For single falls, seven of the eight studies reported a statistically significant difference in the risk of falls between ethnic groups, generally showing the White people tend to fall more than Black and Asian older adults, but did not differ from Hispanics. When observing differences in recurrent falls for the two studies in which this was reported, there was again a reduced risk of falling observed for Asian older adults compared to White in the study of Kwan et al. [38].

## Discussion

This systematic review was limited to only those studies in which fall prevalence was compared between two or more ethnic groups in an attempt to increase the heterogeneity of study design. Studies in which fall prevalence was only reported for a single ethnic group were excluded. However, the wide range of countries in which the studies were carried out, the ethnic groups observed and the differing methodologies used all gave substantial variability to the data.

This variability is evident in the wide range of fall prevalence reported, which ranged from 2.9 to 44.5%. In order to synthesise the data from these multiple studies, a meta-analysis was carried out, using a random-effects model due to the variability of the data. This analysis showed that differences were apparent between the reported fall rates of Asian, Hispanic, Black and White populations, listed here from lowest to highest fall prevalence. This observation was confirmed by unadjusted measures of fall risk, which suggested that White people tend to fall more than other ethnic groups. Even when adjusted for a wide range of contributing factors, White populations had a higher risk of falling than other ethnic groups, both for single and recurrent falls. This is an interesting finding, as the majority of these studies were in the USA where African-American populations have poorer health and living conditions than White Americans in the same area [49], and yet when their risk of falling was adjusted for these inequalities, it was still lower than that for the White older adults. This is also contrary to other age-related conditions such as frailty, in which higher rates of frailty have been reported for African Americans in the USA [50, 51].

There are many potential reasons for the differences observed in these studies. It has been shown that there may be a difference in attitudes to fall risk and participation in risk-taking behaviours between Asian and White groups [38]. Lower fall rates in Chinese groups may be due to greater fear of falling as evidenced by their higher scores in FES-I tests, as well as different cultural behaviours such as greater use of walking sticks. These two factors could result in lower levels of risk-taking behaviours. In addition, increasing fall prevalence with increasing age may affect results in different countries and ethnic groups due to differences in local life expectancy.

In reality, differences in fall prevalence are probably due to a complex interaction of factors including culturally specific behaviours and beliefs, general health characteristics and sociodemographic elements. Culturally specific behaviours may include differences such as those who wish to avoid losing face or showing weakness associated with older age [52], compared with those who are more willing to accept assistance [38]. Health beliefs could involve issues such as having a fatalistic attitude towards falls and potential prevention interventions [53, 54]. Health issues may include chronic illnesses, functional impairments including visual problems or walking difficulties, or common geriatric conditions such as cognitive impairments [52]. BMI is also a risk factor for falls as those with high BMI measures often show altered gait patterns, and postural instabilities that make it difficult to recover from a perturbation [55]. The most important sociodemographic elements for falls are sex and age [56, 57]. All these issues have considerable impacts on fall prevalence and may influence the results either by directly causing differences in the prevalence of falls, or by contributing to differences in how falls are perceived and reported by members of different ethnic groups.

The variability in this study was its main limitation. Heterogeneity was quite high, limiting general conclusions, but this is not surprising given factors such as the disparities within the general groups used. For example, the group termed Asian included Japanese, Chinese, Filipino, and ‘Asian’. These nationalities are all inherently very different, with differences in all the individual factors discussed above as contributing to differences in fall prevalence.

The studies included were carried out in different countries, and with varying methodologies, which naturally cause variance. For example, study design included retrospective data, prospective data and EMR data. Most studies used a retrospective design of between 12 and 24 months. However, older adults frequently have difficulty remembering falls, whether due to having forgotten the fall, or a denial of the fall due to a desire to hide signs of frailty [58–60]. Recall of falls is generally better if the fall was serious and the person suffered a significant injury [58, 60], but if the injuries were minor, they too are easily forgotten [59]. Therefore, data gathered retrospectively may not be reliable.

The sample sizes used in the different studies also varied greatly. From studies using EMR data of 5,510,341 individuals [45], to small studies containing only 114 [43]. These extremes could have very different effects on the results of individual studies, with smaller sample sizes failing to identify relevant effects, and larger ones finding significant differences that are insubstantial. However, the use of a meta-analysis in this paper allowed a single estimate to be obtained for each ethnic group. Even though the larger studies using survey or EMR data were not included in the meta-analysis, the largest study in this analysis with 17,784 individuals [32], still differed greatly from the smallest indicated above.

The covariates used to adjust the data also showed considerable variation. Some studies only adjusted for basic variables such as age, race, study site and body mass index [31], while others adjusted for numerous factors such as age, gender, education, income, neighbourhood deprivation, living alone, health (being overweight, inactivity, alcohol consumption, perceived health, hearing, sight, mobility limitations, multi-morbidity, loneliness, depression) [32]. Studies in which more covariates are adjusted for increases the validity of the findings where any differences in fall prevalence between ethnicities remain. The studies in this paper showed that differences in ethnic groups remained even when ten or more covariates were included in the analysis, showing that there are differences in fall rates due to ethnicity.

The key finding of this study is that fall prevalence differs between ethnic groups, even after adjusting for multiple covariates, which underlines the importance of moving away from a ‘one size fits all’ approach to Public Health. Falling is a significant issue for older adults which carries considerable cost on both the personal and financial front. By identifying the most at-risk groups, resources can be targeted to where they are most needed, such as providing education and fall prevention interventions to those identified as being at risk of falls, ideally before a fall occurs. By appreciating racial and ethnic differences in fall prevalence, there can also be an equal appreciation of the different barriers and requirements of fall prevention interventions for different ethnic groups. The proposal of more ethnically tailored responses to these public health challenges may provide the answer to the low adherence of certain groups to interventions involving physical activity. Further research is needed to indicate exactly how fall prevention interventions could be better tailored to the needs of different ethnic groups, particularly in multicultural societies.

## Conclusion

Differences in fall prevalence do appear to exist between different ethnic groups. Further research is required to determine the reasons for these differences, and to increase the amount of information available on fall rates of different ethnic groups.

## Data Availability

Not applicable.
